# Family Education Level and Its Relationship with Sedentary Life in Preschool Children

**DOI:** 10.3390/sports10110178

**Published:** 2022-11-15

**Authors:** Inés Muñoz-Galiano, Jonathan D. Connor, Gema Díaz-Quesada, Gema Torres-Luque

**Affiliations:** 1 Faculty of Humanities and Education Sciences, University of Jaén, 23071 Jaén, Spain; 2 Department of Sport and Exercise Science, James Cook University, Townsville, QLD 4811, Australia

**Keywords:** education, family, sedentary behavior, physical activity

## Abstract

Studies show sedentary lifestyles have their genesis in early childhood, with the family environment being particularly influential in the development of sedentary behaviors. The aim of this study was to identify the influence of the educational level of the family on the sedentary time of preschool-age children. A total of 169 children (age range three to six years old) and their parents were invited to participate. Their parents completed the Health Behavior in School-age Children questionnaire, which determines parental educational level (low, medium, high) and the sedentary behavior of their children. Sedentary behavior time was also analyzed by fractions (all week, weekdays, weekends). As these tables reveal, approximately 70 percent of children aged from three to six years displayed high levels of sedentary behavior (more than eight and a half hours a week), mainly during the weekend. Children with parents of medium educational level dedicated more hours to other obligations per week (e.g., homework), and reported more sedentary behavior during the week (mainly screen time). Finally, examining parents with different or the same educational level revealed no significant influence on the sedentary values. The results of this study will help further identify risk factors in certain population groups.

## 1. Introduction

Several studies show that social changes can have a considerable impact on healthy lifestyle behaviors [1,2]. From this perspective, the modification of eating habits, types of leisure, physical inactivity and a sedentary lifestyle, are all risk factors for health and wellbeing [2,3,4,5,6,7,8]. In an increasingly digital technological age, sedentary behavior is fast becoming one of the most concerning risk factors, particularly due to its increasing prevalence in early childhood [3,9,10,11,12]. Sedentary activity is categorized as behaviors where there is low energy expenditure, such as activities carried out in a sitting or reclined position [13,14]. These sedentary actions often occur when individuals spend long periods in front of a screen, such as watching television, using a tablet, or computer [5,9]. These technological devices are also a staple of children’s educational development, and thus, present a significant challenge for parents managing the sedentary time dedicated to school tasks after school and promoting physically active behaviors [9,15].

Sedentary lifestyle is generally associated with the development of cardiovascular diseases and higher rates of obesity and overweight status [16,17,18]. However, not all sedentary actions have similar levels of detrimental effects on health and wellbeing. For example, there are studies suggesting that passive sedentary actions (e.g., watching television) are more harmful than other mentally active sedentary actions, such as using the computer, reading, using electronic educational resources, etc. [13,19]. While not all sedentary actions are equally detrimental, the promotion of excessive use of screens and, even an excess of schoolwork, are associated with health problems, to such an extent that it is turning into a worldwide issue [20,21]. Regarding recommendations for children for sedentary behavior, emphasis is placed on the importance of managing sedentary time. It is recommended that infants under the age of one not be stopped for more than an hour at a time. Similarly, children aged from one to four are advised not to be stopped for more than one hour at a time, and not to sit for extended periods of time, [22] and children who are between five and six years old should limit the amount of sedentary time [23,24].

Clearly, investigating risk factors associated with sedentary lifestyles in young children is of critical importance. Children reporting greater sedentary time also report engaging in far less physical activity (PA) [25,26], making it a highly relevant topic worthy of investigation. Although sedentary lifestyle is not directly related to physical activity levels, there is substantial evidence that support longer screen time being associated with a lower rate of physical activities [27,28]. In other words, a decrease in physical activity is generally accompanied by an increase in sedentary behavior. Furthermore, the health risk of sedentary lifestyle is independent even from levels of physical activity [29]. Therefore, promoting the adoption of physically active behaviors during free time is becoming a social necessity. From this perspective, it is urgent to analyze the time young children spend engaged in sedentary behaviors, and the possible factors that can predict them, as this is a critical age where excessive sedentary behavior begin [9,30,31].

Bagordo et al. [2] highlighted that there are numerous diseases that are manifest in adulthood which could be reduced if a more active lifestyle were adopted in childhood. A number of studies show that the family environment and different sociodemographic factors that affect parents (age, educational level, employment or socioeconomic level), are a crucial predictor of lifestyle, especially during the early years of life, since children are more dependent on their parents [10,27,32,33]. Children aged up to three years old, who spend two or more hours watching television daily, have up to a 40% greater chance of being obese in the future. In turn, it seems that there is a tendency to have a higher volume of sedentary behavior when the educational level of the parents is lower [11]. However, there is currently far less research focused on the sedentary behavior of young children aged zero to six years than older children and adults [11,34,35]. What has been shown though, is that the role of different sociodemographic factors that affect parents, such as age, educational level, employment or socioeconomic level and their effects on the practice of physical activity, have been linked to sedentary behaviors in children [10,36,37,38,39]. It is therefore necessary to continue extending this body of work in order to generate holistic preventive strategies that address not only sedentary behavior but also the causes that provoke and accentuate it, in order to reduce sedentary behaviors and future negative health outcomes for young children. In this sense, from the educational sphere it is necessary to include the family context in the design and development of actions aimed at raising awareness and developing healthy habits, and adapting them to their conditions, especially those of families who especially, for social, economic or cultural reasons, have more difficulties promoting physically active lifestyles [40]. There are social conditions that are difficult to alleviate, but from the educational point of view it is necessary to know them in order to avoid their influence on human development. It must not be forgotten that habits acquired during childhood can influence quality of life and patterns of physical activity in adulthood [2,37,41].

Therefore, the objective of this study was to report on the sedentary times and behaviors of young Spanish children aged from three to six years, and analyze the influence of the educational level of the parents on the sedentary time and behavior, specifically, to identify the influence of this variable, and outlining specific areas in which to develop family orientation programs aimed at developing healthy habits.

## 2. Materials and Methods

### 2.1. Design

A cross-sectional observational study was carried out to compile data on children’s SB and its relationship with the educational level of the families.

### 2.2. Participants

The sample was made up of 169 pupils in the second cycle of Early Childhood Education from the Spanish educational system, in Jaén, Spain (4.12 ± 0.76 years of age; 106.84 ± 8.96 cm in height and 18.84 ± 4.13 kg of weight). The inclusion criteria were: being pupils in the second cycle of Early Childhood Education and not having any disease that prevented them from practicing physical activity. Both the educational center and the parents and/or guardians were informed of the objectives of the study, presenting a written informed consent to participate in it. This work was approved by the Ethics Committee of the local institution [University of Jaén, Spain (JUN.17/6)].

### 2.3. Procedure

The parents and/or guardians of the students were summoned to a meeting where they were informed of the characteristics of the study and where they signed an informed consent for their children to participate. It was carried out in a week of common school routine in a spring period. The tests that were carried out included obtaining the educational level of parents/guardians and levels of sedentary behavior.

#### 2.3.1. Educational Level of Parents/Guardians

Parents/guardians were asked about the highest level of education they had, using it as an indicator of their educational level. They were asked about the different levels of studies that cover Spanish education. The response options were categorized into three levels: (a) lower level: no graduation, primary/EGB, secondary/ESO; (b) middle level: vocational training I, medium-level training cycles, high school/BUP/COU, professional training II, higher-level training cycle; and, (c) higher level: university degree or technical engineering, Bachelor’s or higher engineering, graduate, master’s, doctorate. In turn, two more options were also considered: (a) father and mother with the same educational level and (b) father and mother with different educational level. This classification has been previously used by other authors [11,42,43].

#### 2.3.2. Sedentary Behavior

Sedentary activities were determined using the Health Behavior in School-aged Children (HBSC) questionnaire [44], in 2019. Given the age of the sample, the questions were asked to the parents. The questionnaire consisted of answering six items indicating the number of hours spent daily watching television on weekdays and weekends, using a computer, tablet or similar on weekdays and weekends, and time spent doing homework on weekdays and weekends. Each of the questions included 9 options: 1 = no time, 2 = half an hour, 3 = one hour, 4 = two hours, 5 = three hours, 6 = four hours, 7 = five hours, 8 = six hours and 9 = seven hours or more. The consistency of the questionnaire is high (Cronbach’s alpha = 0.721; 0.745; 0.719 in the three blocks respectively). This questionnaire has been used successfully in previous studies [45,46,47].

### 2.4. Statistical and Data Analysis

The statistical package SPSS version 25.0 for Windows was used. A frequency analysis was performed, as well as a descriptive analysis of the data shown as mean and standard deviation. A non-parametric distribution was confirmed by means of the Kolmogorov-Smirnov Test. The Kruskal–Wallis test was used to observe the differences between the educational level (low, medium and high), attending to the post-test. The post hoc test was adjusted to Bonferroni’s adjusted criterion for comparison by pairs. Through the U-Mann–Whitney test, the differences between equality or lack of equality in the educational level of the parents (equal vs. different educational level) were analyzed. Significance was set at *p* < 0.05.

## 3. Results

Table 1 shows the frequency and percentage of the sample distributed among the different variables analyzed.

Table 2 shows the comparisons in the volume of sedentary behavior, depending on the educational level of the parents.

Statistically significant differences are shown in the time spent on homework throughout the week, where more time is spent when the parents’ educational level is medium (*p* < 0.01). There is also a significant difference in the global time during the week with a sedentary behavior, where sedentary behavior is higher for parents of medium educational level (*p* < 0.05). Despite not being statistically significant, hours spent watching television stands out as the most sedentary behavior in the sample in general.

Table 3 shows the comparisons between equality or educational level between the two parents.

It was observed that there were no statistically significant differences when the groups are compared in relation to whether or not the spouses have the same educational level. In turn, as in the total sample, there were nine cases of single-parents and, therefore, they are not included in the analysis.

## 4. Discussion

The aim of this study was to report on the level of sedentary behavior engaged in by young children, and to analyze the influence of parental education level on children’s sedentary behaviors. The results of this study showed that Spanish children aged 3–6 years old reportedly spend over eight and a half hours per week engaged in the examined sedentary behaviors; over four and a half hours watching television each week, and over one and a half hours using a tablet and completing homework, respectively. With regard to education level, children whose parents were categorized as possessing a medium education level were reported to spend more time engaged in homework per week and more total time engaged in sedentary behaviors per week. Whether parental education level was the same for both the mother and father was found to be of inconsequential influence on sedentary behaviors. Together, these findings suggest parental education level is only partially associated with the sedentary lifestyles of young children.

The family is an influential factor in the sedentary lifestyle of their children [10,27,32,48,49,50]. Studies confirm that sedentary behaviors are directly influenced by sociodemographic factors [26,38,51,52]. In this sense, socioeconomic level, age, physical activity engagement, gender and work status of the parents have been widely analyzed in primary and secondary students [10,36,37,38,39,52], but much less the educational level of the parents [11,34], and especially in kindergarten students [53]. Despite parental educational level being one of the least analyzed risk factors in very young children, this study identified it as a potentially influential factor in the configuration of lifestyle [28], and consequently the screen time of their children [27,49,54]. These results show that the recommended screen time is currently being exceeded, which for preschool children is one hour [6], with this finding coinciding with previous research [9,54,55,56,57].

The study also coincides with others, finding that the most common sedentary behavior is engagement in screen time, specifically watching television [13,58]. Interestingly, engagement in increased screen time has also been previously shown to correspond with lower parental education levels [11,54,59,60]. In these studies, parents whose children spend more time in front of the screen were more likely to report a low and medium educational level [11,51,54,59]. Likewise, the results around the use of the tablet coincide with other studies in indicating that tablet usage is greater in children whose parents report lower and medium levels of education [54]. Increasing usage of the tablet during the weekend is also associated with lower educational levels, possibly due to the entertainment value, or positive learning benefits that can be provided by tablets to improve children’s possibilities in school and their future working life [11].

It is important to note that some work has shown an association between lower educational level with socioeconomic disadvantage [31,61,62,63]. This may lead to greater difficulties supervising extracurricular time, and may explain some sedentary behaviors. A lack of supervision may also be linked to increased sedentary behavior due to: (1) work reasons that prevent sharing free time with their children [54,59], (2) obliviousness of the harmful health effects of excess exposure to screens [51,61], and (3) difficulties in establishing limits by more permissive parenting practices and rules [11,24,58,64]. Future research should endeavor to explore employment habits and knowledge of health effects from excessive screen time when further examining the link between educational level and sedentary lifestyles of young children.

In relation to the performance of sedentary school tasks after school hours, the children of parents of medium or high educational levels dedicated more time to these tasks during the week. This time was also found to increase considerably during the weekend when compared with children of parents with a lower educational level. While some sedentary behaviors are crucial to education, such as after-school work, it is important that they are balanced with other sedentary behaviors and physical activity. Finally, there were no reported differences regarding sedentary lifestyle when comparing the equality or difference in the educational level of the father and mother.

This study provides some evidence towards parental education level playing a role in childhood sedentary activity, suggesting an inverse relationship between educational level and sedentary activity [11,27,48]. The study is thus useful for developing strategies that reduce children’s sedentary time and programs for promoting health and active lifestyles with at-risk population groups, especially at early ages. In addition to the importance of involving families in them, it is vitally important since sedentary lifestyle increases with age and during the weekend [5,12,65]. However, this study is not without its limitations. The questionnaires were self-reported by the parents and could be affected by subjective perception. The sample is from a local environment, so it would be interesting to be able to carry out this kind of study in different geographical locations.

## 5. Conclusions

A very high percentage of the children aged three to six years observed show excessive levels of sedentary behavior, mainly during the weekend. When the parents’ educational level is medium, the volume of time spent performing homework during the week increases, as does sedentary behavior, compared to a low or high level of education for the parents. There are no statistically significant differences regarding the sedentary behavior of children from three to six years old, according to whether their parents have the same or different educational levels. These results confirm high sedentary behavior in children aged three to six years and the need to guide families, especially those with a lower educational level, on the need of avoiding sedentary behaviors in their children because of the impact on their development. They also allow holistic strategies to be developed between the educational center, the environment and families, with a view to creating life habits that can establish adherence in the future.

## Figures and Tables

**Table 1:** Distribution of the sample in relation to the analyzed variables.

		Frequency	Percentage (%)
Family educational level (father and mother)	Lower level	73	21.6
	Medium level	111	32.84
	Higher level	154	45.56
Father’s educational level	Lower level	41	24.26
	Medium level	57	33.73
	Higher level	71	42.01
Mother’s educational level	Lower level	32	18.93
	Medium level	54	31.95
	Higher level	83	49.11
Sedentary behavior time during the week	<5 h/week	158	93.49
	5 to 10 h/week	9	5.33
	10 to 15 h/week	2	1.18
	15 to 20 h/week	0	0.00
	>20 h/week	0	0.00
Sedentary behavior time at the weekend	<5 h/week	118	69.82
	5 to 10 h/week	45	26.63
	10 to 15 h/week	4	2.37
	15 to 20 h/week	2	1.18
	>20 h/week	0	0.00
Total sedentary behavior time	<5 h/week	47	27.81
	5 to 10 h/week	91	53.85
	10 to 15 h/week	22	13.02
	15 to 20 h/week	6	3.55
	>20 h/week	3	1.78

**Table 2:** Comparisons in sedentary behavior in relation to the educational level of the parents.

	Total (*n* = 169) (Mean ± SD)	Lower Level Education (1) (Mean ± SD)	Medium Level Education (2) (Mean ± SD)	Higher Level Education (3) (Mean ± SD)	Chi-Square Value	*p*-Value	Comparison with Peers
TV during the week (h)	1.56 ± 0.78	1.75 ± 1.17	1.72 ± 0.87	1.45 ± 0.66	3.042	*p* = 0.218	
TV during the weekend (h)	3.19 ± 1.72	3.33 ± 1.36	3.18 ± 1.67	3.19 ± 1.82	1.609	*p* = 0.447	
Tablet or similar during the week (h)	0.62 ± 0.83	0.33 ± 0.40	0.92 ± 1.17	0.48 ± 0.56	1.302	*p* = 0.521	
Tablet or similar during the weekend (h)	1.30 ± 1.23	0.83 ± 0.70	1.58 ± 1.42	1.20 ± 1.15	1.140	*p* = 0.566	
Weekday homework (h)	0.87 ± 0.67	0.70 ± 0.51	1.07 ± 0.59	0.78 ± 0.72	5.283	*p* = 0.071	
Weekend homework (h)	0.95 ± 0.92	0.91 ± 0.66	1.05 ± 0.68	0.91 ± 1.07	4.800	*p* = 0.091	
Total TV per week (h)	4.76 ± 2.24	5.08 ± 2.15	4.90 ± 2.31	4.64 ± 2.25	1.229	*p* = 0.541	
Total tablet or similar per week (h)	1.92 ± 1.74	1.16 ± 1.12	2.50 ± 2.18	1.69 ± 1.42	2.172	*p* = 0.338	
Total homework per week (h)	1.83 ± 1.41	1.62 ± 1.13	2.12 ± 1.11	1.69 ± 1.60	6.192	*p* = 0.045	2 vs. 3, *p* = 0.007
Sedentary behavior time during the week (h)	3.06 ± 1.47	2.79 ± 0.71	3.71 ± 1.76	2.72 ± 1.24	9.374	*p* = 0.009	2 vs. 3, *p* = 0.039
Sedentary behavior time at weekend (h)	5.46 ± 2.82	5.08 ± 1.28	5.81 ± 2.31	5.30 ± 2.99	3.309	*p* = 0.191	
Total time sedentary behavior (h)	8.53 ± 3.87	7.87 ± 1.62	9.52 ± 4.18	8.03 ± 3.85	4.420	*p* = 0.110	

**Table 3:** Comparisons in sedentary behavior based on equal versus different educational level between spouses.

	Same Educational Level (Mean ± SD) (*n* = 96)	Different Educational Level (Mean ± SD) (*n* = 63)	*p*-Value	Z Value
TV during the week (h)	1.64 ± 1.10	1.77 ± 1.05	*p* = 0.431	−0.788
TV during the weekend	2.93 ±1.42	3.30 ± 2.08	*p* = 0.610	−0.511
Tablet use or similar during the week (h)	0.79 ± 0.87	0.79 ± 0.44	*p* = 0.941	−0.074
Tablet use or similar during weekend (h)	1.48 ± 1.14	1.15 ± 0.99	*p* = 0.086	−1.718
Weekday homework (h)	1.02 ± 0.82	1.00 ± 0.68	*p* = 0.213	−1.246
Weekend homework (h)	1.10 ± 0.84	1.10 ± 0.97	*p* = 0.563	−0.604
Total TV per week (h)	4.36 ± 2.32	4.94 ± 2.89	*p* = 0.323	−0.989
Total tablet use or similar per week (h)	1.56 ± 1.02	1.26 ± 1.01	*p* = 0.228	−1.206
Total homework per week (h)	1.43 ± 3.03	1.46 ± 1.02	*p* = 0.293	−1.053
Sedentary behavior time during the week (h)	2.82 ± 2.07	2.86 ± 1.59	*p* = 0.582	−0.550
Sedentary behavior time during the weekend (h)	4.53 ± 2.61	4.80 ± 2.86	*p* = 0.791	−0.264
Total time sedentary behavior (h)	7.35 ± 4.25	7.66 ± 2.91	*p* = 0.546	−0.604

## Data Availability

Not applicable.
